# Adaptive significance of affiliative behaviour differs between sexes in a wild reptile population

**DOI:** 10.1098/rspb.2023.0805

**Published:** 2023-06-28

**Authors:** C. Delmé, N. Jackson, B. Class, K. Strickland, D. A. Potvin, C. H. Frère

**Affiliations:** ^1^ School of Science, Technology and Engineering, University of the Sunshine Coast, Queensland, Australia; ^2^ School of Biological Sciences, University of Queensland, Brisbane, Queensland, Australia; ^3^ Ludwig-Maximilians-Universität München, Munich, Germany; ^4^ Institute of Ecology and Evolution, School of Biological Sciences, University of Edinburgh, Edinburgh, UK

**Keywords:** social behaviour, behavioural syndromes, fitness, eastern water dragon, multivariate mixed models, wild population

## Abstract

In recent years, we have begun to appreciate that social behaviours might exhibit repeatable among-individual variation. Such behavioural traits may even covary and have critical evolutionary implications. Importantly, some social behaviours such as aggressiveness have been shown to provide fitness benefits, including higher reproductive success and survival. However, fitness consequences of affiliative behaviours, especially between or among sexes, can be more challenging to establish. Using a longitudinal behavioural dataset (2014–2021) collected on eastern water dragons (*Intellagama lesueurii*), we investigated whether various aspects of affiliative behaviour (i) were repeatable across years, (ii) covaried with each other at the among-individual level, and (iii) influenced individuals' fitness. In particular, we considered affiliative behaviours towards opposite-sex and same-sex conspecifics separately. We found that social traits were repeatable and covaried with each other similarly for both sexes. More notably, we found that male reproductive success was positively correlated with the number of female associates and the proportion of time spent with females, while females’ reproductive success was not correlated with any of the measured social behaviour metrics. Overall, these findings suggest that selection may be acting differently on social behaviour of male and female eastern water dragons.

## 1. Introduction

Understanding how affiliative social behaviours influence individual fitness is key to exploring the evolution of social behaviour. The theory of social evolution posits that sociality, i.e. the degree to which individuals in a population associate, should evolve when the net benefits of social interactions outweigh the costs [1]. Beneficial affiliative social interactions may emerge in the form of grooming, cooperative resource acquisition and predator vigilance, while risks include disease transmission, social conflict and inbreeding [1]. Over the last 15 years, empirical research has provided strong support for the adaptive significance of affiliative behaviour across the animal kingdom [2]. In fact, multiple aspects of individuals' affiliative behaviour (e.g. number of associates, quality and stability of associations) have been shown to vary consistently among individuals, and have been linked to a variety of fitness advantages including increased reproductive success and survival in primates [3–5], rodents, ungulates, dolphins [2,3] and even reptiles [4].

Although it is well-known that individuals can affiliate with one another in multiple different ways [5], whether or how these different social behaviours correlate within individuals is still under investigation [6]. One theory posits that all social or affiliative behaviours reflect the same process at the individual-level, indicating the presence of a latent ‘sociality trait’ [7] (also referred to as a ‘behavioural syndrome’ [6,8]). Current evidence supports the idea that sociality can be considered a heritable phenotypic trait, although the extent to which it is repeatable and/or plastic appears to vary across individuals and species [7]. Regardless of whether they can be generalized, as with any potentially linked traits, the existence of behavioural syndromes may impose evolutionary constraints on the attributes that comprise them owing to phenotypic or genetic covariances [9].

An important source of among-individual (co)variation in social behaviours is sex. Indeed, sex differences have previously been identified in a number of social behaviours, including social tendency [8,10,11], sociability [12], number of associates [8,10,13] and social network position [10]. Additionally, as males and females are often subject to different selective pressures, their social behaviour towards same-sex or opposite-sex individuals—and any fitness consequences thereof—is likely to differ. For example, competing for access to females has resulted in the evolution of male-male social alliances in species such as dolphins and baboons (multiple species: [14,15]). By contrast, female orangutans (*Pongo pygmaeus abelii)* and macaques (*Macaca assamensis)* may form strong relationships with males to avoid sexual harassment [16,17]. Furthermore, female-female (but not male-female) social bonds have been shown to increase reproductive success in bottlenose dolphins (*Tursiops aduncus*) [18], and longevity in chacma baboons (*Papio ursinus*) [19]. However, despite growing interest in sex-specific social behaviours, we still know little about how the adaptive significance of social behaviours—for either sex—varies according to the nature of the social relationships (i.e. same-sex versus opposite-sex).

Here, we used a longitudinal behavioural dataset (2014–2021) collected on eastern water dragons (*Intellagama lesueurii*), combined with parentage analyses and multivariate mixed models, to investigate among-individual covariation in multiple measures of sex-specific social association and their fitness consequences. The eastern water dragon is a long-lived, semi-aquatic diurnal lizard, occurring along the east coast of Australia [20]. Individuals reach sexual maturity around three years of age, with females being polyandrous and generally laying 4–12 eggs twice a year [21]. Eastern water dragons display strong sexual dimorphism and dichromatism, where males exhibit red ventral coloration and are larger than females [20]. Eastern water dragons are a gregarious reptile with fission-fusion-like social systems. Individuals foster both preferences and avoidance for certain conspecifics which can vary across space [22] and local densities [23]. Sexes also show differences in their social strategies whereby males are repeatable in their social tendency (i.e. proportion of time an individual spent within 1.85 m of conspecifics), number of associates and centrality [10]; whereas females are socially more plastic and will modify their degree of social tendency based on the composition of their local density [24]. Furthermore, social dominance has been shown to influence individual fitness in this population, where reproductive success increased with social dominance for inbred males, while the opposite was true for outbred males [25].

Within each sex, we investigated whether different measures of affiliative social behaviour with a given sex, and the density of conspecifics of each sex within an individual's home range, (i) were repeatable, (ii) correlated with each other at the among-individual level, and (iii) correlated with fitness. Because eastern water dragons have previously been found to exhibit phenotypic correlations between social behaviours [10] and sex-dependent social strategies [23,26], we expected to find different patterns of covariation between the sexes. More importantly, we expected that behaviours reflecting higher levels of sociality would be adaptive (i.e. correlate with increased reproductive success) given the fitness benefits of affiliative behaviour across the animal kingdom [2].

## 2. Methods

### (a) Data collection

We used data collected as part of an ongoing behavioural study that started in 2010 of an urban population of eastern water dragons inhabiting Roma Street Parkland (RSP), Brisbane, Australia (27°27′46′ S, 153°1′11′ E). The RSP population has an estimated size of 336 individuals which are highly habituated to human presence [27]. The parkland spans approximately 16 ha and is composed of various habitats differing in size, complexity and heterogeneity of structure, vegetation and access to water [28].

### (b) Behavioural data

We carried out behavioural surveys twice a day (7.30–10.30 and 13.00–16.00), by following a transect which covers approximately 85% of the known population, three days a week from September to April (when dragons are most active [10]) each year (hereafter referred to as a season). For each individual, we took a photo of their left and/or right profile, along with their GPS location which was later used to calculate spatial proximity and define social associations (see ‘Social traits’ section). An individual's sex was assigned based on sexual dimorphism and dichromatism [20]. We then recorded the individual's immediate behaviour at the time of the observation. Behaviours included a spectrum from resting to agonism (e.g. head bobs, tail slaps, arm waves, chasin and physical confrontations; see [25] for more details).

Profile photographs were used to identify individuals using a previously established method for this population [28]. We employed an interactive identification software (I3S Spot, v. 4.0.2), comparing individual facial patterns with an established photo library. Those without a match were considered new individuals.

### (c) Genetic data

Animals were caught during sessions independent from the behavioural surveys. Each season all possible adult individuals were caught using a lassoing technique. A photo was taken of each individual for identification upon capture. Blood samples were collected using the ventral tail caudal venepuncture technique [29], while tissue samples consisted of collecting the tip of the tail [26]. DNA was extracted using DNeasy extraction kits (Qiagen) [25] and sequenced using restriction-associated digest methods at Diversity Arrays Technology, Canberra, using proprietary DArTcap technology. This method applies a selective step after complexity reduction to genotype specific markers from DArTseq representations [30], which is a reduced representation sequencing approach, similar to RAD-sequencing. Single nucleotide polymorphisms (SNPs) were identified using the DArTsoftS pipeline [31,32]. This resulted in a total of 6425 SNPs prior to filtering, including 510 juveniles (which were not included in analyses, but were used to inform parentage assignment) and 775 male and female adult individuals.

### (d) Social traits

#### (i) Social associations

Social associations were defined as pairs of individuals socially tolerating each other in close spatial proximity (1.85 m). Details of these measurements can be found in the electronic supplementary material, methods.

For each year, the following aspects of social behaviour were measured: (i) proportion of time an individual spent within 1.85 m of conspecifics (i.e. social tendency), (ii) number of associates (i.e. degree), and (iii) average half-weight association index (hereafter referred to as HWI; see the electronic supplementary material, methods for exact calculations). HWI estimates the strength of social associations using the weighted proportion of time pairs of individuals spend together. Each social behaviour was calculated using opposite-sex and same-sex associations separately. All social behaviours were calculated in individuals' core home ranges (detailed under ‘*Conspecific density’* methods), and only individuals with at least 30 sightings per year were included in analyses. Although a minimum of 20 sightings is required to produce stable estimates of social behaviour in this species [10], we chose to use a more conservative threshold as this did no drastically impact data retention and allows for greater confidence in estimates.

### (e) Conspecific density

We calculated the density of conspecifics within each individual's core home range (HR_50_). Core home ranges were estimated using the kernel utilization distribution method in the adehabitatHR package [33], and a previously optimized smoothing parameter of 7 m was used to control for the amount of variation around density estimates [28]. The coordinates of the 50% probability contour were then extracted to obtain HR_50_. The core home range was chosen as this represents individuals' primary territory and is where most social associations occur [22,27]. Density of conspecifics (hereafter density) was estimated for individuals with at least 25 sightings, as this is the minimum number required to calculate robust home range estimates [27]. Once estimated, density of conspecifics was divided into two categories, namely opposite-sex density (i.e. containing overlapping individuals of opposite-sex only) and same-sex density (i.e. containing overlapping individuals of same-sex only).

### (f) Fitness

Fitness was defined as reproductive success, which was estimated by calculating the number of offspring that survived to adulthood (hereafter referred to as ‘number of offspring’) [34]. To estimate the number of offspring, parentage assignments were performed in the Sequoia package, using SNP data, within the R statistical environment [35]. Sequoia uses a fast, heuristic hill-climbing algorithm, which has been shown to result in low error and high assignment rates with only a few hundred highly informative, unlinked SNPS [35]. SNP data for use in parentage assignment were obtained by applying the following filters: (i) read depth of at least five reads for homozygote genotypes, (ii) individual call rate greater than or equal to 70%, (iii) SNPs call rate greater than or equal to 99%, and (iv) proportion of technical replicate assay pairs for which the marker score was consistently greater than or equal to 99%. Minor allele frequency threshold (MAF) was chosen by undertaking sensitivity analyses, where we generated a set of files in which SNPs were filtered using different MAF threshold values (range: 0.38–0.47). We then performed parentage assignments for each dataset and compared the outputs.

Parentage assignments obtained from Sequoia were validated in two ways. First, we compared them to known maternities resulting from field observations of nesting events [36]. Second, genetic relatedness was calculated using the maximum-likelihood dyadic relatedness estimator [37] in Coancestry [38]. We then plotted parentage assignments against relatedness estimates (electronic supplementary material, figure S1). SNPs used for the relatedness analysis were obtained from less stringent filtering (individual call rate ≥80%, SNP call rate ≥95%, MAF ≥ 0.05, proportion of technical replicate assay pairs ≥98%), as a higher density of SNPs may increase the accuracy of relatedness estimation [39]. This resulted in a total number of 2100 SNPs to estimate relatedness.

The best MAF value, as indicated by the highest parentage assignment and absence of disagreements with field observations was MAF ≥ 0.43, which resulted in a sample size of 179 SNPs used for parentage assignment. From the 775 adult individuals included in the parentage assignment, 239 dams and 296 sires were assigned surviving adult offspring. When validating assignments against known maternities, 98 from the 106 were correctly assigned (92%), while six mother-offspring pairs were not assigned (6%) and two were mismatched (2%). These mismatches, however, could be the result of mis-assigned mothers from field observations, owing to nest-sharing events (C. Delmé 2019-2022, personal observation). While it is possible that we may have missed genotyping individuals that would have been assigned to other parents, this likelihood is low (overall genetic sampling rate from the entire population was 80%).

### (g) Statistical analyses

All analyses were performed using multivariate linear mixed-effect models fitted in MCMCglmm [40,41] within the R statistical environment [42]. For each sex separately, we estimated: (i) the repeatability of social behaviours, (ii) correlations between social behaviours, and (iii) correlations between social behaviours and reproductive success (i.e. number of offspring). Repeatability was estimated separately in males and females, as sex differences in repeatability of social behaviour have previously been found in our population [10]. Social tendency, mean HWI and density were centred at the population mean value and standardized to units of one standard deviation. All measures of central tendency for posterior densities refer to mean values.

### (h) Repeatability of social behaviours

Based on our criteria of at least 30 sightings, our raw dataset of 39 270 observations within individual core home ranges translated to 556 yearly social measures (*n* female = 347; *n* male = 209) from 133 females and 92 males, of which 141 had repeated observations across years.

To investigate whether social behaviours consistently differed among individuals, we fitted multivariate models for males and females, for each opposite-sex and same-sex yearly measures of individuals' affiliative behaviour (four models in total). In all models, the number of sightings per individual and number of years individuals had been sexually mature (i.e. age) were included as fixed effects. Individual identity and year were fitted as random effects. Residuals and random effects were fitted with heterogeneous variance and co-variance was fixed using the ‘idh’ function and model intercepts were fit per trait. Models were fitted default normal priors for fixed effects and weakly informative priors for residual and random effect variance components (*V* = *n* and nu = 0.002, where *n* = number of response variables). To improve model fit, a higher degree of belief parameter was used for residuals (nu = 0.02), this did not alter the model estimates and hence these results are reported herein. We further ran all models with parameter expanded priors for all random and residual variance components to ensure that prior specifications did not impact results. Given estimates did not change, all results reported are with priors specified and *V* = *n* and nu = 0.002 for random effects and nu = 0.02 for residuals. For both opposite-sex and same-sex social behaviour, degree was analysed with a Poisson distribution, while social tendency, mean HWI and density were analysed using a Gaussian distribution. Models were run with a burn-in of 2 × 10^3^, 3 × 10^6^ iterations and a thinning interval of 1000. To assess model fit we inspected trace plots along with effective sample sizes (greater than 1000) and sampling autocorrelation (less than 0.1). For each model, we considered fixed effects significant from zero where the 95% credible intervals of the posterior distribution did not overlap with zero. We then estimated repeatability using the following:

$$R=\frac{\mathrm{VI}}{V_{I}\;+\;V_{R}},$$

where *V*_I_ represented the among-individual variance, *V*_R_ corresponded to the within-individual variance, and *V*_I_ + *V*_R_ described the total phenotypic variance in the population [43,44]. Variance estimates from multivariate repeatability models can be found in the electronic supplementary material. The repeatability of traits estimated with a non-Gaussian error distribution were transformed from latent scale to the data scale using the QGglmm package in R [45]. To test for differences between the sexes we used the R function pd_to_p in the R package *BayestestR* to generate *p*-values for significance.

### (i) Correlations between social behaviours

As with multivariate models for repeatability, among-individual correlations were investigated for each sex separately. We investigated correlations (i) across social behaviours of each category (e.g. between opposite-sex social tendency, opposite-sex degree, opposite-sex preferences and opposite-sex density); and (ii) between opposite-sex and same-sex categories of each of our social behaviours (e.g. opposite-sex social tendency and same-sex social tendency).

To investigate correlations across social behaviours of each category (males same-sex traits, males opposite-sex traits, females same-sex traits and females opposite-sex traits) we adjusted the previously described repeatability models to include a CORGH variance structure to estimate individual-level correlations between opposite-sex and same-sex social behaviours and their 95% credible intervals using marginal posterior modes (equivalent to best linear unbiased predictions (BLUPs) and for simplicity is hence referred to as). By specifying CORGH variance structures with prior specification V as the number of response variables, it allows us to estimate individual-level correlations and not among individual variances in social traits (which are fixed to 1). Correlation models were run with 2 × 10^6^ iterations, with weakly informative priors (*V* = *n*, nu = 0.002), with all other model specifications and diagnostics otherwise run as described above.

By contrast, when investigating correlations between opposite-sex and same-sex categories of social behaviours, bivariate models were performed. Bivariate models were run with 2 × 10^5^ iterations, with weakly informative priors (*V* = *n*, nu = 0.002), with all other model specifications and diagnostics otherwise run as with multivariate correlation models described above.

### (j) Behavioural correlations with reproductive success

As indicated by Houslay & Wilson [46], multivariate models do not require that each variable must be a repeated measure, thus allowing for the investigation of correlations between repeatedly measured variables and other variables with only one observation per individual, such as reproductive success [46]. This procedure allows the estimation of the uncertainty of individual-specific effects for the repeatedly measured trait. Therefore, in our study, multivariate models were fitted for each sex separately to investigate the correlations between individuals' social behaviour (repeated measures of same-sex social tendency, same-sex degree, same-sex mean HWI, same-sex density) and the number of offspring. In all models, individual identity and year were included as random effects, while the number of sightings per individual and number of years individuals had been sexually mature were included as fixed effects. Given that heterozygosity and social dominance have previously been shown to influence reproductive success in our study species [25], these two variables were also included as fixed effects. Heterozygosity was estimated using internal relatedness, representing one of the best performing heterozygosity estimators [47], while social dominance was calculated as the proportion of aggressive displays per year [27]. Individuals which morphologically reached adult sizes [20] in the 2018–2019 season or later were excluded from analyses of fitness because their offspring were unlikely to have been genotyped and included in parentage assignment. Because we had a single measure of fitness (i.e. number of offspring) for each individual, this response had no within-individual variance. Therefore, residual and individual level random effect variances were fixed to 1. Model priors, residual variance structures, distributions, burn-in iterations, thinning intervals and iterations for each model were specified as with previous multivariate correlation models. Fitness was analysed using a Poisson distribution. Given the large number of zeros in the fitness dataset, models were further run with zero-inflated Poisson distribution for fitness. For these models, convergence was not achieved as indicated by a high degree of autocorrelation and hence we report models using Poisson distribution for fitness. Differences between sexes was determined as per the repeatability models above.

Our measures of reproductive success were limited to individuals which (i) had at least 30 sightings per year in the behavioural surveys, (ii) remained in the genetic data post-filtering, and (iii) were adults capable of producing adult offspring within the study timeframe. Hence, correlations between social behaviours and fitness were investigated using 537 observations across 101 female and 203 male individuals. Of these, 106 male and 168 had at least one offspring, while the remaining 262 individuals were assigned zero offspring.

## 3. Results

Social tendency varied from 0 to 0.90 (males: 0–0.81, females: 0–0.90), degree from 0 to 20 (males: 0–20, females: 0–16), mean HWI (strength of social associations) from 0 to 0.67 (males: 0–31, females: 0–0.67) and density from 0 to 0.14 (males: 0–0.10, females: 0–0.14).

### (a) Repeatability of social behaviours

All social behaviours except for opposite-sex HWI in both males and females, exhibited between-individual variance estimates that were significantly different from zero. We found that the proportion of time an individual (male or female) spent with conspecifics (social tendency) of either sex was moderately repeatable (table 1). Similarly, the density of conspecifics within the core home range was also moderately repeatable, regardless of the sex of the focal individual or surrounding conspecifics (table 1). For the number of associates (degree), repeatability estimates varied from low to moderate in both sexes (table 1).

**Table 1.** Repeatability estimates and 95% credible interval from the univariate models performed on each opposite-sex and same-sex social behaviour in males and females separately. (Note: ‘OS’ indicates that social behaviours (e.g. social tendency, degree, HWI or density) involved opposite-sex social associations only, while ‘SS’ involved same-sex social associations only.)

	females		males	
social behaviour	latent	raw	latent	raw
social tendency OS	—	0.29 [0.17;0.4]	—	0.41 [0.23;0.55]
social tendency SS	—	0.42 [0.28;0.55]	—	0.35 [0.21;0.54]
degree OS	0.85 [0.45;0.96]	0.11 [0.04;0.2]	0.5 [0;0.77]	0.08 [0;0.2]
degree SS	0.81 [0.38;0.94]	0.2 [0.11;0.34]	0.58 [0.21;0.91]	0.22 [0.07;0.49]
HWI OS	—	0 [0;0.15]	—	0.09 [0;0.24]
HWI SS	—	0.32 [0.19;0.43]	—	0.21 [0.02;0.37]
density OS	—	0.48 [0.33;0.62]	—	0.31 [0.11;0.46]
density SS	—	0.52 [0.31;0.64]	—	0.39 [0.18;0.53]

### (b) Correlations between social behaviours

When we considered our four social traits, we found that most were positively correlated with one another in both males and females, regardless of whether the social trait was measured between same-sex or opposite-sex interactions (table 2). The exceptions to this both centred around association strength (HWI), which was not statistically correlated with either the number of associates (degree) nor density of conspecifics in their core home range (table 2). This was consistent across both males and females, regardless of which sex with which they were interacting. The strongest correlation was identified as existing between number of associates (degree) and density of conspecifics for male-male behaviours.

**Table 2.** Correlation values and 95% credible interval from multivariate models investigating correlations across social behaviours for males and females separately. (Mean posterior distribution values are reported with 95% credible intervals and estimates for which the 95% credible interval did not overlap zero are highlighted in bold. Note: ‘OS’ indicates that social behaviours (e.g. social tendency, degree, preferences or density) involved opposite-sex social associations only, while ‘SS’ involved same-sex social associations only.)

social behaviour 1	social behaviour 2	females	males
social tendency OS	degree OS	**0.44 [0.20;0.67]**	**0.58 [0.35;0.80]**
social tendency OS	HWI OS	**0.42 [0.20;0.64]**	**0.53 [0.30;0.71]**
HWI OS	degree OS	−0.28 [−0.56;−0.02]	0.05 [−0.29;0.40]
density OS	social tendency OS	**0.43 [0.19;0.63]**	**0.45 [0.16;0.72]**
density OS	degree OS	**0.57 [0.34;0.76]**	**0.52 [0.24;0.77]**
density OS	HWI OS	−0.17 [−0.43;0.10]	0.06 [−0.25;0.43]
social tendency SS	degree SS	**0.59 [0.37;0.80]**	**0.6 [0.36;0.81]**
social tendency SS	HWI SS	**0.47 [0.26;0.67]**	**0.53 [0.23;0.81]**
HWI SS	degree SS	−0.22 [−0.53;0.14]	0.33 [0;0.65]
density SS	social tendency SS	**0.51 [0.31;0.71]**	**0.48 [0.20;0.75]**
density SS	degree SS	**0.57 [0.35;0.78]**	**0.65 [0.29;0.75]**
density SS	HWI SS	0.10 [−0.16;0.36]	0.31 [0;0.63]

Examinations of whether sexes were demonstrating consistency in their social behaviours whether interacting with same-sex or opposite-sex individuals found that the number of associates correlated, as well as density (table 3). In other words, if either males or females had more same-sex associates, they were also more likely to have more opposite-sex associates. Similarly, if individuals had more opposite-sex conspecifics in their core home range, they were also more likely to have same-sex conspecifics present as well. Interestingly, the other opposite-sex and same-sex trait correlations (social tendency and HWI) were not significantly different from zero. This is, association strengths between same-sex individuals did not correlate with the association strength between opposite-sex individuals, and social tendency or time spent with one sex did not correlate with time spent with the other sex, either positively or negatively.

**Table 3.** Correlation values and their 95% credible interval from bivariate models investigating correlations within social behaviours for males and females separately. (Mean posterior distribution values are reported with 95% credible intervals and estimates for which the 95% credible interval did not overlap zero are highlighted in bold. Note: ‘OS’ indicates that social behaviours (e.g. social tendency, degree, mean HWI or density) involved opposite-sex social associations only, while ‘SS’ involved same-sex social associations only.)

	females	males
social tendency OS - SS	0.21 [−0.07;0.45]	0.18 [−0.15;0.51]
degree OS - SS	**0.34 [0.07;0.63]**	**0.41 [0.08;0.71]**
HWI OS - SS	0.24 [−0.01;0.48]	0.16 [−0.18;0.48]
density OS - SS	**0.67 [0.49;0.81]**	**0.56 [0.31;0.79]**

### (c) Reproductive success

**Figure 1.**
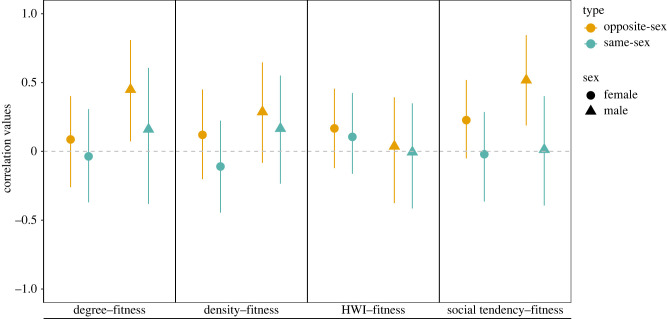
Correlation values and 95% credible intervals of the posterior distribution obtained from multivariate models investigating correlations between reproductive success and social behaviours in males and females separately.

**Figure 2.**
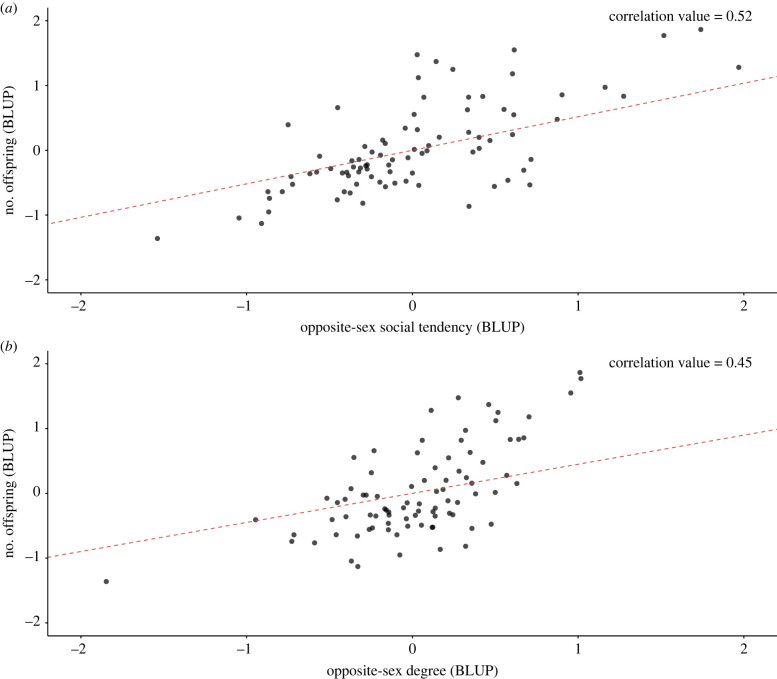
Correlation between (*a*) males opposite-sex social tendency and (*b*) males opposite-sex degree (mean-centred and standardized) and number of offspring from multivariate models. BLUPs were extracted from the model and are represented here.

The number of offspring per individual in our dataset varied from 0 to 9 (males: 0–9, females 0–9). Based on our multivariate models, we found sex-dependent effects of social behaviour on reproductive success (table 4). Males with higher opposite-sex social tendency and opposite-sex degree values had a higher number of offspring (opposite-sex social tendency-fitness correlation value = 0.52; opposite-sex degree- fitness correlation value = 0.45; table 4, figures 1 and 2). By contrast, for females, none of our measures of social behaviour for opposite-sex or same-sex categories exhibited a correlation with the number of offspring that was significantly different from zero.

**Table 4.** Correlation values and 95% credible interval for the posterior distribution from multivariate models investigating correlations between same-sex and opposite-sex social behaviours and fitness. (Estimates for which the 95% credible interval did not overlap zero are highlighted in bold. Note: ‘OS’ indicates that social behaviours (e.g. social tendency, degree, preferences or density) involved opposite-sex social associations only, while ‘SS’’ involved same-sex social associations only.)

	females	males
social tendency OS	0.20 [−0.05;0.52]	**0.52 [0.19;0.84]**
degree OS	0.08 [−0.26;0.40]	**0.45 [0.07;0.81]**
HWI OS	0.16 [−0.12;0.46]	0.04 [−0.38;0.39]
density OS	0.10 [−0.20;0.45]	0.28 [−0.08;0.65]
social tendency SS	−0.02 [−0.36;0.29]	0.01 [−0.39;0.40]
degree SS	−0.04 [−0.37;0.31]	0.16 [−0.38;0.61]
HWI SS	0.11 [−0.31;0.42]	0.16 [−0.38;0.61]
density SS	0.11 [−0.16;0.22]	0.17 [−0.23;0.55]

## 4. Discussion

In this study, we used a covariance partitioning framework to investigate the adaptive significance of social behaviour in eastern water dragons. Our work represents one of the very few studies [19,48] demonstrating that individually repeatable social behaviours have an impact on individual fitness. Specifically, we found that both sexes exhibited consistent among-individual differences in social behaviours. We then demonstrated that most social behaviours correlated positively with each other in both sexes, but that the extent of these correlations tended to vary when considering the sex of individuals’ associates (i.e. opposite-sex or same-sex). Last, and arguably most importantly, we showed that the proportion of time spent with opposite-sex conspecifics (social tendency) and the number of opposite-sex associates (degree) was positively correlated to fitness in males but not females.

Eastern water dragons have previously been found to exhibit sex differences in the repeatability of a range of social behaviours, including social tendency, centrality and number of associates within an individual's entire home range, with males being more repeatable than females [10]. Here, we built upon this previous work by investigating affiliative behaviour at a finer scale, that is, by considering opposite-sex and same-sex behaviour within an individual's primary territory (i.e. core home range [22,27]). In contrast to Strickland & Frère [10], we found that, in both males and females, all social behaviours exhibited consistent among-individual differences, with little differences between the sexes. This may be because, in our study, we investigated social interactions occurring within individuals' primary territories, whereas Strickland & Frère [10] considered individual's entire home ranges. Eastern water dragons have previously been shown to modify their social behaviour across space, whereby individuals increase their number of social preferences and avoidances within their primary territory [22]. Taken together, these contrasting results suggest that patterns of consistent among-individual differences can vary across an individuals' home range, and that social behaviours may be more repeatable within an individual's primary territory.

Importantly, most social behaviours were positively correlated with each other, regardless of the sex of either the focal individual or its associates. From an animal personality perspective, a suite of (repeatable) behaviours that covary with each other at the among-individual level signals a ‘behavioural syndrome’ [49], and thus may reflect different measurements of an underlying social ‘trait’. Such correlations may also capture genetic associations between behaviours and therefore indicate coevolution [49]. While other studies have also found correlations between social traits, results are highly variable, and there are known issues with comparing different species and studies, especially those that use differing measurements or behaviours [7]. Despite this, our positive correlations across the social measures social tendency, degree, and density, conserved between the sexes, may indicate that they all capture a similar aspect of an individual's sociality.

Regarding sex-specific correlations between social behaviours, these results contrast with our expectations in this specific study system, but align with earlier studies in other species. We did not find that suites of behavioural traits differed between the sexes, despite eastern water dragons exhibiting strong sex-bias in social behaviour. For instance, females associate more with both sexes than males do with other males [26], and the number of same-sex preferences is greater in females compared to males [22]. In line with our findings, a previous study on delicate skinks (*Lampropholis delicata*) found that females were more social and tended to be faster explorers than males, while both sexes exhibited a similar behavioural syndrome between activity, exploration and sociability [11]. Altogether, this highlights that while sexes may differ in how they interact with conspecifics in their social environment, social behavioural traits can nonetheless covary similarly between the sexes. More importantly, our work represents one of the few studies using covariance partitioning frameworks to investigate sex differences in behavioural correlations, especially in wild populations [8,50,51].

Sex-specific selection can represent a major source of variation in selective pressures [52]. This has previously been demonstrated in the context of sociality in bighorn sheep, for which centrality increased survival in both sexes, while only increasing reproductive success in females [48]. Here, while opposite-sex social tendency and degree was positively correlated to reproductive success in males, we found that selection may also act on sex-specific behaviours in eastern water dragons. Our findings indicate that males benefit from the quantity of their social associations. This is probably a case of increased mating opportunity relative to the number of females that are social associates [53,54]. Interestingly, this echoes recent findings that house sparrows (*Passer domesticus*) with more opposite-sex associates gain within-year fitness advantages [55]. However, this pattern appeared to exist for both sexes in sparrows, whereas only male dragons appeared to benefit from these particular social traits. Because fitness benefits of affiliative behaviours differed between males and females, while their covariance structure did not, interesting research avenues would include examining whether these behaviours have genetic underpinnings that are conserved between the sexes and whether this could constrain the evolution of sociality in this species (i.e. sexual conflict). Sexual conflict arising from differences in selection on social behaviour has, to our knowledge, not yet been explored, and would constitute an interesting new research question at the intersection of behavioural and evolutionary biology.

To conclude, this study expands our knowledge about social behaviour and its fitness consequences and provides rare sex-specific estimates of among-individual correlations between social behaviours and between social behaviour and fitness in a wild population. This study also aims to encourage further research on sex-specific social behaviour and their fitness consequences, and to promote the use of (co)variance partitioning approaches, which, to date, have been rarely applied when investigating correlations [56] and selection on repeatedly measured labile traits [57]. Since co-variation of social traits is probably population or species specific, we recommend that further research be completed on other taxa and environmental contexts in order to more fully understand social strategies across the animal kingdom.

## Data Availability

The data are provided in Dryad Data Repository https://doi.org/10.5061/dryad.ttdz08m23 [58]. The R code is attached as a supplementary file. Additional information is provided in the electronic supplementary material [59].
